# Putting patients first: when home-based care staff prioritise loyalty to patients above the system and themselves. An ethnographic study

**DOI:** 10.1186/s12910-024-01094-0

**Published:** 2024-09-11

**Authors:** Cecilie Knagenhjelm Hertzberg, Morten Magelssen, Anne Kari Tolo Heggestad

**Affiliations:** Faculty of Medicine, Institute of Health and Society, Centre for Medical Ethics, University of Oslo, Gaustadalléen 21, Oslo, 0349 Norway

**Keywords:** Home-based care, Ethnography, Ideal of good work, Ethics of care, Mature care, Moral courage

## Abstract

**Background:**

The growing number of older people worldwide poses challenges for health policy, particularly in the Global North, where policymakers increasingly expect seniors to live and receive care at home. However, healthcare professionals, particularly in home-based care, face dilemmas between adhering to care ideals and meeting external demands. Although they strive to uphold ethical care standards, they must deal with patients’ needs, cooperation with colleagues and management guidelines. Home-based care is an essential part of healthcare services in Norway, but staff struggle with high patient numbers and time management. This article focuses on how staff deal with ethical challenges related to contextual and organisational constraints.

**Methods:**

An ethnographic fieldwork in three municipalities in South-East Norway. The first author conducted three to four months of participant observation in each municipality. In addition, she conducted in-depth interviews with key informants in two municipalities and a focus group interview with seven home-based care workers in one municipality. The data was analysed by using a reflexive thematic analysis.

**Results:**

Staff in home-based care are frequently more loyal to the patient than to the system and to their own needs. To provide good care, all informants disregarded the patient’s formal decision, i.e. they provided more care than the formalised decision stipulated. To prioritise beneficence to patients, informants also disregarded some of the rules applicable in home-based care. In addition, staff accepted risks to their own safety and health to provide care in the patient’s home.

**Conclusion:**

The loyalty of home-based care staff to their patients can go beyond their loyalty to the rules of the system and even their own safety. This commitment might be attributed to a sense of doing meaningful work, to providing relationship-based and individualised care, and to strong moral courage. However, the staff’s emphasis on flexibility and individualised care also brings challenges related to unclear boundaries related to patient care.

**Supplementary Information:**

The online version contains supplementary material available at 10.1186/s12910-024-01094-0.

## Introduction

The increasing number of older people globally poses challenges for health policy and service development. In the Global North, policymakers emphasise that people should live at home for as long as possible [1, 2]. At the same time, nurses and other healthcare professionals seem to be caught between strong normative ideals of care and the external demands of patients, other professionals, and management. The healthcare professionals advocate principles and standards that determine how patients should be cared for. These ideals, rooted in ethical principles and societal expectations, serve as a guide for healthcare professionals to provide high-quality care [3]. Staff are under pressure and expectations from many sources, including patient needs and preferences, requirements for collaboration with other healthcare professionals, and management directives. This leads to a complex balancing act for healthcare professionals as they attempt to maintain their commitment to quality care while managing the practical realities and expectations imposed on them by patients, colleagues and management.

Employees providing home-based care work in the patient’s home, often working alone as a “visitor” in the patient’s home [4]. However, the patient’s home may not be equipped or adapted for medical care. The patient’s health situation is often complex and varied, including somatic and mental health conditions. In addition, many patients live with a partner or children.

Previous studies have shown that home-based care workers have difficulties adapting care to patients’ needs [5–8]. Tailoring care to each patient is seen as a core value for home-based care workers, but the organisation of care has made it difficult to fulfil this value [1, 6–8]. According to research on Norwegian home-based care by Olsen et al., staff in home-based care expressed that they must be a “balancing artist”, which means that they are faced with competing and conflicting demands i.e., patient needs versus the organisational requirements. Staff attempt to manage care resources and try to care for as many patients as possible [1]. Furthermore, Norwegian researchers within nursing ethics, Tønnessen, Nortvedt and Førde found in their research on home-based care and priority setting that care often lacks flexibility, personalisation and responsiveness to circumstances that are critical for optimal patient care. They describe that home-based care is driven by the clock and not by individual needs, therefore staff must prioritise medical and physiological needs rather than taking a holistic approach to the patient [6]. This is consistent with the result of Norwegian researcher Hestevik et al. who studied challenges faced by healthcare professionals in the specific case of providing personalised nutritional care to patients living at home. The study highlights the difficulties staff face in meeting patients’ specific nutritional needs. Hestevik et al. argue that the organisation of care has become rigid and standardised, which means that the needs and preferences of each individual patient are no longer taken into account. The healthcare professionals in the study recognised that it is important to look at nutritional problems holistically rather than seeing them as an isolated problem but that there was a lack of time to individualise nutritional care when caring for patients in their home which can be generalised to home-based care in general [5]. It is crucial to gain insight into the impact the system and organisation has on healthcare professionals in home-based care.

## Theoretical framework and key concepts

We discuss our findings in light of the theory of *an ethics of care* and the concepts of *good work*, and *moral courage*.

While traditional ethical theories, such as deontology and consequentialism, emphasise human beings as rational and independent, an *ethics of care* emphasises emotions, interdependency, and relations [9–11]. This means that emotions may play an important role in moral judgement, and that there lies an ethical imperative in our interdependency and relationships with others [10]. In addition, an ethics of care focuses on the actual situation with the patient rather than universal and general norms or rules. This means that the patient’s needs in the actual situation take centre stage and care must be adapted accordingly [12–15].

The ideal of *good work* is a concept developed by the psychiatrists Howard Gardner, Mihaly Csikszentmihalhi and William Damon [16]. They developed the concept because they were struggling to find a balance between high-level performance and social responsibility, i.e. between ethics and excellence. This led to the development of the concept of *good work*, which refers to professional quality work that benefits society as a whole. They also explored what promotes or hinders good work in today’s context. In their book *Good Work: When Excellence and Ethics Meet*, they examine what it means to do “good work”. The authors use examples from the professions within genetics and journalism when discussing the concept, but we found that it also applies to the work of health professionals. Gardner et al. ask what strategies enable people to maintain a moral and ethical standard at work. The concept is developed empirically by examining how professionals continue to do “good work” at a time when commitments to profitability and efficiency can threaten professional and ethical ideals. Furthermore, they argue that “good work should be something to which one should be committed according to one’s profession. The employee skills, accountability and ethical considerations are essential components for employees to feel that they are excelling in their work [16]. To perform “good work” is essential when working as a healthcare professional as it also fosters engagement and perseverance [8]. We have identified a connection between the ethics of care and ‘good work’ in home-based care, as both are focussed on doing what is best for the patient and not for the system.

We have also discussed our finding in light of the concept of *moral courage*. To have moral courage can be described as the determination to uphold one’s moral values and act, even in the face of possible negative outcomes or consequences [17–19]. Moral courage is linked to both the ethics of care and *good work*, because ultimately moral courage means doing what is best for patients, despite the challenges faced by home-based care staff.

### Home-based care in Norway

Home-based care is the largest primary healthcare service in Norway [20]. The number of patients is steadily increasing, patients are discharged earlier, and treatment is continued at home. Home-based *services* are state-subsidised services for all Norwegian citizens who lives at home, it include home-based care, physiotherapy, occupational therapy, and mental health care. In this context, we focus on home-based care, a service that emphasises basic medical care for patients, e.g. help with medication administration, nutrition, wound care, morning and night care, palliative care, cancer care, and so on.

In Norwegian home-based care, patient care is regulated by formal decisions to which staff must adhere. This formal decision determines the type of help the patient needs and can be adjusted if their needs change. Typically, the process of receiving home-based care is initiated by the general practitioner or a relative who helps the patient to apply. The municipality then assesses the patient’s needs and determines the type of help needed, resulting in a formal decision [21]. The staff in home-based care consist of nurses, auxiliary nurses and unskilled workers. Home-based care is a general term for all healthcare services provided in the patient’s home. It is healthcare and care at the interface between daily life and the provision of public services [22]. Patients requiring home-based care are found in all stages and situations of life.

The biggest change in the municipal health sector in the last decade has been in home-based care. This is mainly due to the implementation of the coordination reform, which means, among other things, that home-based care has been given a new and more comprehensive role. In addition, there has been an enormous increase in patient numbers, which have quintupled between 1992 and today. The large number of patients in home-based care makes continuity difficult [22, 23]. At the same time, home-based care staff must take a holistic approach to the patient, i.e. focus on all factors that influence the patient’s health. This means that home-based care staff must provide individual care within a very narrow organisational and contextual framework [22].

### Aim

The project aimed to explore the ethical challenges faced by home-based care staff, and how they handle these challenges. We understand ethical challenges as challenges in which values may come into conflict. Handling ethical challenges is about prioritising which values are the most important [24]. In this article, we focus on how employees deal with ethical challenges related to contextual and organisational constraints.

## Method

This paper is based on an ethnographic study conducted in home-based care in three municipalities in the South- Eastern part of Norway from September 2020 to November 2021. The first author (CKH) conducted the fieldwork which involved participant observation and in-depth interviews, she was in each municipality for three to four months, however, the number of days in the field varied due to the pandemic (Table 1).

Participant observation allows researchers to better understand people’s behaviour by participating in their everyday lives and witnessing real-life situations in their natural environment [25, 26]. Previous studies have shown that it can be difficult for staff to put ethical challenges into words [27, 28]. Through participant observation, CKH was able to discover and explore the ethical challenges that might not have been put into words by the informants in an interview. Combining participant observation and interviews allowed for the findings from the observations to be followed up through the interviews and vice versa. Together, these methods provided a rich and comprehensive set of data [26, 29].

**Table 1:** Fieldwork

Municipality	Time	Days of participant observation
1	September-November 2020	8 (56 h)
2	March-May 2021	15 (92 h)
3	August-November 2021	18 (122 h)

### Participant observation

During the participant observation, CKH accompanied two employees in each municipality who acted as key informants (Table 2). She assisted with patient care, such as helping to prepare meals, talking to the patient, and assisting with hygiene when the patient needed the help of two people (typically for patients who required extensive bedside care). CKH also attended meetings and breaks, etc. CKH observed how the key informants interacted with the patients and how they handled challenging situations. Through the informal conversations with the informants during car rides, meal breaks and meetings, she got the chance to talk about topics in a casual way and listen to how staff interact and discuss their patients and challenging situations. CKH wrote fieldnotes on an iPad during the participant observation; together with other data these were stored on a secure server. The fieldnotes consisted of: observations of home-based care and patient care; conversations with key informants; other staff (who had signed a consent form); managers; patients and their relatives; and conversations during breaks and meetings. In addition, the fieldnotes included CKH´s reflections on her role in the field and her thoughts. The fieldnotes were written “on the go”, i.e. in the car, during breaks or while waiting for a key informant.

Due to the pandemic and restrictions, participant observation was limited to eight days in Municipality 1. In Municipalities 2 and 3, COVID-19 still influenced daily life, but at this stage there were fewer restrictions. Thus, participant observation was conducted for 15 days in Municipality 2 and 18 days in Municipality 3. CKH followed an observation guide which was built on the research questions throughout the fieldwork. Topics were: (a) Context of home-based care; (b) Observations and what happens when staff are “out there”; (c) Observations before and after working with a patient (Additional File 1).

### Interviews

CKH and AKTH performed a focus group interview in Municipality 1 with seven home-based care staff and key informants (Table 3). Focus group interviews are a good way to elicit different perspectives from informants, and they foster an open atmosphere in which the thoughts of all informants are valuable and heard [30]. Due to COVID-19, we were not able to conduct focus group interviews in the two other municipalities. In Municipalities 2 and 3, CKH conducted in-depth interviews with the two key informants from each municipality simultaneously (Table 2). She used one interview guide for the focus group interview and one for the joint interviews in Municipalities 2 and 3. The themes of the interview guides, which are built on the research questions, were: (a) Working in home care; (b) Ethical challenges or/and difficult situations; (c) Communication and coordination; (d) Caring for patients and (e) Ethics support. In addition to asking questions from the interview guide, CKH addressed observations that had occurred during the fieldwork e.g., patients, situations, conversation, etc. (Additional File 2). All interviews lasted for about 1–2 h. The interviews were recorded and transcribed by CKH.

**Table 2:** Overview of Key informants

Municipality:	Pseudonym:	Profession:	Working years:
1	Anne	Palliative nurse	6 to 10 years
	Line	Auxiliary nurse	16 to 20 years
2	Emilia	Nurse	1 to 5 years
	Silje	Psychiatric auxiliary nurse	6 to 10 years
3	Elise	Nurse	1 to 5 years
	Ida	Nurse	16 to 20 years

**Table 3:** Overview of informants in focus group interview (municipality 1)

Pseudonym:	Profession:	Working years:
Marie	Nurse	20 to 25 years
Mathilda	Nurse	10 to 14 years
Andrea	Nurse	1 to 5 years
Helga	Nurse	16 to 20 years
Sonja	Auxiliary nurse	16 to 20 years
Anne (Key informant)	Palliative nurse	6 to 10 years
Line (Key informant)	Auxiliary nurse	16 to 20 years

### The three municipalities

The three municipalities differed both geographically and in types of housing. Municipality 1 was rural; the houses were spread over a large area. Many of the patients’ homes were not age or illness-appropriate, meaning there were no lifts, steep stairs, and high thresholds. The second municipality was rural and urban. Some patients lived in apartments, others in houses. CKH spent most of the fieldwork time in the living facility Betwixt-house (fictive name) for old people, meaning it was age or illness-appropriate. Municipality 3 was urban, and patients lived in flats of varying standards. Some had modern and new flats that could be easily adapted to age or illness, e.g., lifts, low thresholds, no stairs, and big bathrooms. While others lived in old flats that were not adapted to age or illness, some lacked facilities such as lifts, bathrooms, toilets, hot water, etc.

### The participants

Six key informants, two in each municipality, were recruited by their managers before the fieldwork (Table 2). In addition, CKH spoke to home-based care staff in general, i.e., in meetings or breaks. All home-based care workers were nurses, auxiliary nurses, or unskilled workers.

CKH visited homes where patients with different needs, diagnoses and care requirements lived. Forty-five patients were recruited based on their willingness to participate, capacity to consent and health status (Table 4). It was the key informants listed above who recruited them. As this was ethnographic fieldwork, it was important for CKH to have access to some of the patients so that she could observe the key informants at work with the patients. CKH also conducted individual interviews with patients and their families, but these data are not part of this article.

**Table 4:** Overview of patients

Municipality	Patients recruited	Main health issues:
1	15	Old age; Cancer; Multiple sclerosis; Dementia; Chronic obstructive lung disease; Heart and lung disease; Mental illness; Substance abuse; Liver failure; Diabetes
2	8	
3	22	

### Analyses

In ethnographic research, the researcher translates and interprets while observing. This means that the process of analysis begins with the writing of fieldnotes [31]. Our data consists of fieldnotes from the participant observation and transcribed in-depth interviews with informants.

We used Braun and Clarke´s model of reflexive thematic analysis when analysing fieldnotes and interviews [32]. The advantage of this model is its pragmatic relation to the philosophy of science and methods, which entails flexibility in the process of analysis when combining different methods, and it enabled us to use the same analytic approach when analysing both fieldnotes and interviews. [32]. Researchers within qualitative research emphasise the importance of being reflexive throughout the research [25, 33, 34]. Reflexivity entails critically reflecting on your role as a researcher, your research practice, and how you may influence the data. It also means that the researcher becomes aware of their position or point of view, values and worldview, all of which may influence the research [34]. In this study, the first author was aware that the data collection could be influenced by her bias as a researcher in medical ethics and a privileged Norwegian woman. However, her background was in anthropology, not in healthcare or ethics, thus she had a holistic and open-minded approach throughout the data collection. During the fieldwork, CKH closely observed the interactions between key informants, staff and patients and their strategies for coping with daily challenges. Despite the lack of explicit ethical discourse, CKH interpreted her observations to recognise and understand the ethical dilemmas associated with the staff’s daily practice. All three authors participated in the analysis, although the main analysis was done by the first author. In the initial phase of the analysis during the fieldwork, AKTH and CKH frequently discussed the results and thus found initial codes. When the fieldwork was completed in all municipalities, the authors met several times to identify, discuss, develop, review, and refine patterns and themes. During the process of analysis, they moved back and forth between the six steps described by Braun and Clarke, which are described as follows [34]. (1) Familiarising ourselves with the data: CKH noticed recurring patterns during the fieldwork, these were written in the fieldnotes and raised in the interviews. Furthermore, they were confirmed at a later point by reviewing the transcribed interviews and fieldnotes. (2) Sorting the initial codes: CKH began categorising the data by going through fieldnotes and interview transcripts several times, creating multiple mind maps, colour categorising the fieldnotes and noting themes. At this phase, the authors began to discuss the results together. (3) Exploring themes: The authors discussed the preliminary themes identified from the coding i.e., the patterns CKH had discovered while working with a mind map colour categorising, and systematically organised them. In this phase, the writing process started, and CKH started to formulate the themes. (4) Reviewing themes: The authors reviewed the themes, in relation to the coded data and the overall data set. (5) Defining and naming themes: The three authors jointly refined and named themes and subthemes. The findings were discussed in relation to theory; CKH, AKTH and MM met and discussed this several times. (6) Summarising and writing up: the authors synthesised and revised the text and finalised the article.

### Ethical considerations

This project was assessed by the Norwegian Agency for Shared Services in Education and Research (reference: 980490). The project was exempted from the duty of confidentiality by the Regional Committee for Medical and Health Research Ethics (REC South-East Norway reference: 130005). Written consent was obtained from all participants, and they were informed that they could withdraw at any time during and after fieldwork. Patients were recruited by the key informants of each municipality. The primary researcher met with patients once they signed the consent form.

The researcher must proceed with caution when conducting participant observation of vulnerable people, as many are unable to protect their interests [26]. CKH discussed her role with the key informant before seeing a patient. Both the key informant and CKH always reminded the patient on arrival why she was there and that she could leave if the patient did not feel comfortable. CKH also planned with the patients, e.g., she knew that a patient in Municipality 3 did not want her to come on Thursdays because that was the day the patient showered. All patients who took part in the fieldwork were competent to consent.

## Results

The results are divided into three themes: **Overriding the patient’s formal decisions**; **Loyalty to the patients over the rules**; **Risking own health and safety**. Our findings show that home-based care staff may experience several contextual and organisational constraints and that they go to great lengths to provide the best possible care to patients within these frames. The first theme shows how home-based care staff do not let the patient’s formal decision prevent them from providing individualised and personalised care. The second theme demonstrates how staff break some of the organisational rules to provide care. The final theme shows that home-based care workers sometimes prioritise the needs of patients even over their own safety and health.

### Overriding the patients’ formal decisions

Home-based care staff provided care within a system in which the patient’s formal decision is supposed to determine which care should be given. The formal decision can be seen as a working tool for the staff, outlining all tasks associated with the patients. There were differences between the municipalities in how strictly they implemented and interpreted the formal decisions. However, as it turned out, all key informants prioritised individualised and personalised care over adherence to formal decisions.

When starting their work shift, staff received a list of patients, which also indicated the time allocated to each task. For example, administering medication was estimated to take two minutes and showering ten minutes. However, CKH observed that the key informants often did not adhere to the time allocated in the lists. The patients could have different needs from day to day. Some days they did not need all the care listed in the formal decision, while other days they needed more care than was estimated. Additionally, for several patients, home-based care was their only connection to the outside world, and CKH observed that the key informants in all municipalities provided extra care for these patients.

How the informants dealt with formal decisions seemed to depend on the management and the degree of autonomy of the employees. In Municipalities 1 and 3, the management left the patient work to the employees and trusted them to make the right decisions while they themselves carried out the administrative tasks; the employees had a high degree of autonomy. CKH observed that the key informants in Municipalities 1 and 3 used the patient’s formal decision as a guide rather than a strict scheme to follow. Some of the informants in the focus group interview in Municipality 1 explained:

CKH: I have seen that you have a schedule with how much time each patient has, do you follow that?

Helga: We don’t use it; we don’t care about that (laughter). Nobody cares about the time.

Sonja: (…) We don’t work like that; we take our time. We are good at knowing where to be fast and where to use more time.

Line: …and it may vary, we may use a lot of time with a patient one day and less the other day. You feel your way [i.e., you use discretion].

The key informants in these two municipalities also explained that they thought there was no point in stressing about time. CKH observed that the key informants often offered or asked if the patient needed more help than what was stated in the formal decision, e.g., asking if patients wished to take a shower even if it was not “shower day”.

On the first day of fieldwork in Municipality 3, Ida and CKH had a conversation about this issue:

I ask about the formal decision, and Ida says they don’t really care about that (…) Ida says you cannot work in home-based care if you are hung up on the formal decisions (Fieldnotes, Municipality 3).

CKH also observed that the staff in Municipalities 1 and 3 often switched patients during the shift. This typically happened when one staff member spent more time with a patient and then a colleague took over the care responsibilities for another patient on the list. This flexibility meant that staff could take a more holistic approach to caring for patients who needed more time.

In Municipality 2, management was more involved and exercised more control over the employees; thus, the informants had less autonomy when performing tasks detailed in the formal decision. During meetings, management emphasised the importance of following the patient’s formal decision and that they were not allowed to act outside of it. Sara, the patient coordinator in Municipality 2, explained why it was important that the staff followed the formal decision:

If we don’t look at the service process, how much time they use, then we won’t get resources because then they don’t see [what staff do] at the city hall. Right, everything is statistics the further up [in the system] we get. And to get more people, they have to see that the statistics are right (Interview, Municipality 2).

On several occasions, CKH witnessed staff in Municipality 2 being reprimanded in meetings if they had disregarded the formal decision. The staff were instructed by the manager to set their boundaries, but it was not that easy. As Emilia said: “You can’t just walk away when someone is crying”. Silje and Emilia did not record it if they provided care outside of the formal decision out of fear of being reprimanded by the manager. Silje stated during the interview:

I don’t really care about the documentation thing because you don’t need… I can go home with a clear conscience because I know she’s eating, it’s these simple things because I’ve sat down [with the patient] (Interview, Municipality 2).

### Loyalty to the patients over the rules

Within the home-based care system, staff must adhere to several rules governing the profession as a whole or established at the local organisational level. Concerning the latter, employees must abide by several do’s and don’ts set by each municipality. Such rules may vary from municipality to municipality, but the rules referred to as examples in our findings apply in all three municipalities. Healthcare professionals were not allowed to accept gifts or money from patients unless the gifts were of low value (e.g. a chocolate bar). Furthermore, they were not allowed to accept credit cards or money to go shopping for the patient or to sit down and eat with them. CKH saw that loyalty, empathy and the staff’s desire to act benevolently towards the patients were more important to them than following these rules.

During the fieldwork, CKH observed what we may call a reciprocal relationship between the informants and the patients. Some key informants explained that patients could experience a lot of satisfaction if staff reciprocated their appreciation. In these situations, the key informant had to consider their role as the professional, and their commitment to make the patient feel good about giving back. They also explained that it was difficult to refuse small gifts from patients. Elise, a nurse in Municipality 3, and CKH had a conversation about this:

They often have to say no [to gifts], for example when asked if they want to eat dinner with a patient. But Elise does not say no when she gets a chocolate bar before Christmas. That makes the patients very happy and is a way of showing that they are grateful [for the care] (Fieldnotes, Municipality 3).

Sometimes the professional role of staff and the law were outweighed by the obligation to ensure that patients had everything they needed in their daily lives. For example, staff sometimes accepted money from patients to buy necessities. CKH noticed that this was commonplace in Municipality 3. Several patients who lived in this area were struggling financially while confined to their own homes due to their health conditions and the layout of the housing (e.g., lack of lifts). Another challenge was that many patients did not have relatives to do their shopping for them. Some patients also relied on food provided by the Salvation Army. CKH noticed that Ida and the other staff in this area were aware of the social and economic situation of the patients. They were willing to go to great lengths and break the rules to fulfil not only the care itself but also the basic needs of the patients. For example, Ida helped Thomas, a patient without relatives, to buy a new telephone and a fridge during her workday. It seemed that the staff had a silent agreement that they would provide these services for the patients. This was unique to this area.

In Area Y (Municipality 3), informants regularly experienced that patients did not have enough to eat. An example was Roman, who lived on the sixth floor without a lift in a municipal housing apartment. He had no relatives and could not walk down the many stairs. One day, key informant Ida and CKH came to see him, and he had no food in the fridge. It was Friday and he was not due to get food again until Monday. CKH spontaneously asked if he wanted her to go shopping for him. She thought she could do this as she worked as a researcher, but according to Ida, this was often done by the staff as well. So, CKH accepted some money and went shopping for him. As she walked down the street, she thought that if the money was not enough for what Roman wanted, she would pay the rest herself. Ida later confirmed that she often pays with her own money so that patients would have something to eat; she does not ask for the money back but accepts it if they want to pay it back. After the visit to Roman, Ida and CKH talked about the matter:

As we go on, we talk about patients who cannot afford food and that this can be difficult for staff. Sometimes they have no food in the fridge or no money. The staff are not allowed to buy food for the patients even if they are given cash. And they are absolutely not allowed to accept bank cards. However, Ida buys food for the patients with a clear conscience. She says she will stand by it, even if her manager disagrees. In the past, she has also paid for food for the patients and received money later. They have to eat (Fieldnotes, Municipality 3).

### Risking own health and safety

Key informants and other home-based care workers often put the welfare of patients above their own health and safety. During the fieldwork, CKH observed or heard several descriptions of situations in which care workers accepted risks to themselves, either because the situation itself was dangerous or because they had to take risks to care for the patients.

There were situations where patients preferred to continue living at home even though their living environment was not suitable for care and posed a potential health risk to staff. To accommodate patients’ preferences, staff sometimes had to improvise to provide adequate care. This led to a double-edged situation where patients felt safe at home, with their routines and things, while staff provided care under difficult conditions and risked their health.

The key informant and other staff in Municipality 1 claimed that most homes were not suitable neither for the health care providers to deliver care, nor for frail old people to live, e.g., showers in the bathtub, stairs and high thresholds which made it difficult to move around in the house, and in some cases unhygienic conditions. The informants explained this during a lunch break in Municipality 1:

We went back to the headquarters to have lunch with the others. [During the conversation] it becomes clear that many are dissatisfied with the living situation of the patients. Many of the homes are not set up in a way that sick and frail people can live there. It is not practical for the patient or home-based care. Some examples are toilets in the basement or the shower in the bathtub. That the bed is too small or too low. This makes the work of home-based care more difficult (…). A patient can function well in a hospital or nursing home where everything is facilitated. But at home, there are corners and edges, stairs, bathtubs and so on. This makes life more difficult and uncertain for the patients (Fieldnotes, Municipality 1).

Several patients in Municipality 1 and 3 lived in old houses or flats in which the patients had lived all their lives without modernising them. In some cases, it was difficult for key informants and other home-based care workers to carry out their work, especially when there was no space for auxiliary equipment or the patient did not want to use such equipment. For tasks such as catheterisation or wound care, an adjustable bed would be beneficial for staff as they would not have to bend over the patient and contort their backs. However, several patients did not have an adjustable bed. Some patients said they did not want their home to look like a hospital or they did not have the space. In these situations, the informants provided the care, even if it meant accepting risks to their health.

In most cases, staff in home-based care work alone, which may sometimes put them in uncomfortable or dangerous situations. However, not providing care was not an option even when staff were scared or felt threatened.

Informants reported caring for patients with severe mental illness, substance abuse problems, or possessing various types of weapons. Staff had to traverse dark alleys in troubled neighbourhoods; occasionally experienced sexual harassment, or had to treat patients they would learn belonged to criminal networks.

There are patients here [assisted living facility] that clearly should be admitted somewhere else. They have a patient who is suicidal and aggressive, she should also be admitted. Emilia is afraid of going to her because she has been standing with a knife or a pan (Fieldnotes, Municipality 2).

When they talked about this, the key informants emphasised that this was part of their job. CKH had the impression that in a way they were used to the situation because they were quite at ease talking about cases that would likely scare most people.

I attended a safety course once, and there they thought it was unbelievable that we went in [to these patients] and that we were alone; they would never do so (Focus Group Interview, Municipality 1).

During the focus group interview informants also mentioned that they do not have their last names on the name tag anymore. They also use tricks from working in the psychiatric ward to maintain their safety:

We don’t have scissors or a neckband, we put the car in the right direction [to facilitate swift departures], we don’t let the [the patients] be behind us (…), we don’t wear a ponytail (Focus Group Interview, Municipality 1).

## Discussion

According to our findings, home-based care workers seem to silently protest the system they work within. The staff’s loyalty to the patient’s individual and personalised needs overshadows the strict and rigid formal decisions, rules, and often even their own safety and health. We discuss these findings considering the ideal of “good work”, moral courage, and ethics of care.

### The ideal of “good work”

According to our findings, nurses in home-based care strive to perform what Gardner et al. define as *good work* [16]. Gardner et al. claim that people who do *good work* not only have requisite skills but also reflect on their responsibilities and how their work affects their personal and professional lives. They strive to act responsibly in various areas, including their goals, their relationships and in the world in general. Our findings suggest that staff are “straining” themselves to fulfil their responsibilities as healthcare professionals and that this is affecting their relationship with patients. *Good work* is also characterised by high professional and ethical standards and personal commitment, which implies taking care of professional, social and ethical implications of the different tasks during the day [8, 16]. Such attitudes align with national and international codes of ethics for nurses which emphasise nurses’ primary responsibility to patients and the demonstration of professional values, including respect, justice, responsiveness, care, compassion, empathy, trustworthiness, and integrity [35, 36]. These codes serve as frameworks for ethical practice and decision-making in nursing and are usually consistent with professional standards set by regulatory bodies. Home-based care workers seem to prioritise the effective fulfilment of their tasks and compliance with professional ethics, even if they rarely express ethical considerations explicitly.

Gardner et al. delineate three fundamental aspects of today’s working life. *The mission*, which includes the defining characteristics of the profession; *the standards*, which represent the recognised “best practices” within the profession; and *the identity*, which reflects workers’ integrity and values [16]. Arguably, placing our results in the context of these three aspects sheds light on home care staff’s situation: the *standards* encompass not only the system and the rules but also the concept of professionally sound care. In Norwegian health law, professionally sound care is a requirement enshrined in the Health Personnel Act [37]. Tønnessen et al. have argued that Norwegian home-based care staff struggle, due to resource constraints, to attend to even basic needs of patients [6]. In our study, we find that staff go to significant lengths to take care of such needs. By having developed a professional morality, home-based care workers may judge what is right or wrong when caring for a patient. They also possess some fundamental values that characterise the entire service. These values revolve around the preservation of the patient’s life and health through healing, comfort and disease prevention [38]. In the results, we see that the staff go to great lengths to care for the patients’ individual needs. They break rules, disregard formal decisions, and accept risks to themselves in the process.

### An ethics of care approach in home-based care

We may also say that our informants acted in line with the core tenets of the *ethics of care*. The ethics of care puts the caring relation at its centre [12–14]. Our informants demonstrated this to a great degree by showing loyalty to the person cared for, not the system.

In the ethics of care, the focus is on understanding the situation and not on adhering to general rules and norms, requiring an ability to use moral judgment [15]. It emphasises the importance of adapting to the specific needs of the individual in every situation [13]. As Martinsen claims, sometimes we must deviate from the letter of the law [15]. In our findings, key informants and other staff prioritised responding to patients’ immediate needs over strict adherence to organisational tasks, and tasks seemed to use their moral judgment when assessing patients’ needs. This is the opposite of what Tønnessen et al. found in their study on home-based care and priority setting in 2011 [6]. They found that home-based care staff lack flexibility, personalisation and responsiveness to circumstances and are driven by the clock [6]. CKH did not observe that the key informants refused a patient’s request for additional care but, rather the opposite, to offer more care.

In her study on standardisation in home-based care, Bjørnsdottir found that time allocation was standardised but flexible [39]. Staff recognised the importance of using time with patients, e.g. emotional support and conversation, as this was seen as essential to the patient’s well-being [39]. Our findings also suggest that staff in home-based care provide more care than is set out in the formal decision and take a holistic approach centred on the whole person. This is in line with what Bjørnsdottir found in her study of home-based care in Iceland [39]. For the staff, good care consisted of flexibility and a willingness to adapt the care to the patient’s needs. She for instance found that staff rearranged the patient list among themselves to better meet the patient’s needs, which promoted a good care environment [40]. Potentially, however, this attitude can give rise to a new challenge: a devotion to holistic patient-centred care might make it difficult to determine just how far staff’s responsibilities extend and conceal the real structural deficits in patient support in the healthcare system. This is especially so as several patients experienced extensive and complex healthcare needs and sometimes lived quite destitute lives. The constant endeavour of healthcare staff to go to great lengths in acting altruistically toward their patients can contribute to burnout. This is perhaps evidenced by the higher sick leave rate among employees in the Norwegian Municipal Health Service compared to employees in other areas of the health service, including other municipal services [41, 42].

While the staff’s patient-centred approach involves flexibility and individualised care, we also witnessed that it leads to unclear boundaries as to how far their services should go, which may also be seen as a criticism against an ethics of care framework [3]. Here one may argue that a formal decision, if used with moral discernment, may be of help to set justified limits and avoid overburdening. According to Pettersen and Hem, *mature care* is when the interests of both healthcare workers and the patient are considered [43]. Furthermore, they argue that reciprocity in mature care does not mean that the carer and patient share or exchange equally. However, the amount of care provided shall depend on the situation. A carer who practices mature care can adapt the care to the situation [43]. Based on the results, we will argue that the key informants exercise a kind of mature care, in seeing the situation and the needs of the patients and acting accordingly. Ida, for example, goes further in caring for her patients in Area Y than Elise does for her patients in Area X. Many patients in Area Y had no relatives, and Ida and the rest of the staff working in that area accordingly provided extra care for them. This does not mean that the other key informants provide poorer care. If the patient’s formal decisions and the rules are flexible, then the staff can adapt the care to the situation.

### A caring culture characterised by moral courage

The results emphasise that employees act according to their professional ethics, even if the system and rules may prevent them from doing so. According to Kleemola et al., moral courage is a critical component of nurse’s ethical competence which enables them to act by their ethical beliefs [18]. It may be understood as the ability to rise above fear, to act and to stand up for one’s moral values, even if one risks negative consequences [17–19]. Furthermore, moral courage is characterised by genuine presence, moral integrity, a sense of responsibility, honesty, commitment, perseverance, and a willingness to take personal risks. Moral courage alleviates moral distress, increases the well-being and work commitment of nurses, and thus contributes to better patient care [18, 19]. In light of the results, we would argue that key informants sometimes practised moral courage. The staff showed courage when they rebelled against the system and the organisation, especially in Municipality 2, where they were not allowed to provide care outside the formal decisions. Staff in this municipality risked reprimand if they defied formal decisions, but they stood up for what was most important to them: caring for the patient. The employees demonstrated moral courage by taking risks for the well-being of the patients. This may mean caring for patients in a difficult home environment or potentially dangerous situations. Whilst this is beneficial to patients as they receive the care they need, the benefits to staff may not be obvious. Staff are putting their health and safety at risk for the benefit of patients. This suggests that some patients may be better suited to live in a nursing home or other facility where additional equipment and support are available. We can also ask whether the situations described are sufficiently safe for patients. The political guidelines in Norway stipulate that people live at home for as long as possible [44]. At the same time, healthcare workers must provide professional care to patients living at home [45]. This obligation can be jeopardised if it is no longer safe for the patient to live at home. This raises the question of whether this is moral courage or moral “hubris”, i.e. staff concealing the significant dangers and the recklessness of patients who are too ill to live at home.

The results also highlight the importance of setting certain requirements for patients so that staff feel safe when entering a patient’s home. In addition, the fear of being reprimanded can have a negative impact on the work culture and create an atmosphere of fear.

Tønnessen et al. found that the purchaser-provider model in home-based care, with an emphasis on formal decisions, may lead to rigid and non-individualised care [6]. Our findings on the other hand show that staff can oppose this rigid system and “stopwatch mentality”, and that healthcare personnel may act according to their moral values by prioritising individualised care. This also shows that home-based care may differ between municipalities and that care may depend on different care cultures. According to Rehnsfeldt et al. a culture of care is oriented towards human dignity, where the ethical behaviour of individual carers reflects the importance of this culture [46]. “Slow care”, where quality is placed above quantity, is seen as part of this culture and helps to ensure that people are valued through a caring relationship. The combination of dignified care, ethics and ongoing discussion is critical to providing appropriate care for patients [46, 47]. However, carers in home-based care can face challenges if the culture does not fully support the ideals of dignity and autonomy.

### Strengths and limitations

Our findings are based on fieldwork in three municipalities that differ in many ways but do not represent all home-based care in Norway. Nonetheless, we think our findings may be transferable to similar settings. The informants were recruited by the management of the respective municipality. Thus, there is a possibility that they selected the “best of the best”. In addition, CKH conducted the fieldwork during the time of COVID-19, which affected accessibility, especially in Municipalities 1 and 2. We also know that the presence of CKH in the field may have influenced the informants and the data. However, the researcher’s effect may have diminished over time. Combining interviews with participant observations may also have strengthened the findings.

## Conclusion

The solitude of providing care in the patient’s home leads to some ethical challenges for staff. In this article, we have discussed the fact that home-based care staff are loyal to the individual and personal needs of their patients and that this loyalty takes precedence over the system, the rules and the staff’s own health and safety. Arguably, this trend may hurt the staff in the long term. We have argued that the staff’s commitment to their patients often goes hand in hand with moral courage and is in line with the basic principles of care ethics and professional ethics. While staff’s holistic approach and desire to put the patient at the centre promotes flexibility and individualised care, it also leads to health and safety risks and a lack of clarity about their professional boundaries and responsibilities as home-based care providers. This suggests that the current system may not work as well in home-based care as it does in other areas of healthcare. The rigidity of a formal decision does not fit the context of home-based care and results in staff working more than they should. If the formal decision was just a guideline and there was the option to provide more care if needed, staff would be more flexible without fear of being reprimanded. The system should also give staff the security they need when providing care that could jeopardise their health or safety, i.e., a safe and secure work environment This is a problem that management needs to understand and address by developing strategies to help home-based care staff deal with risky patient situations.

## Electronic supplementary material

Below is the link to the electronic supplementary material.

Supplementary Material 1

Supplementary Material 2

## Data Availability

We are unable to share the data (transcripts and fieldnotes) due to the conditions in theresearch ethics approval: the participants might be identified from the transcripts and they have not consented to sharing of these data.

## References

[1] Olsen CF, Bergland A, Debesay J, Bye A, Langaas AG. Striking a balance: Health care providers’ experiences with home-based, patient-centered care for older people—A meta-synthesis of qualitative studies. Patient Educ Couns. 2019;102(11):1991–2000. 31160128 10.1016/j.pec.2019.05.017

[2] Björnsdóttir K, Ceci C, Purkis M. The ‘right’ place to care for older people: home or institution? Nurs Inq. 2015;22(1):64–73. 23786552 10.1111/nin.12041

[3] Hem MH, Halvorsen K, Nortvedt P. Altruism and mature care: some rival moral considerations in care ethics. Nurs Ethics. 2014;21(7):794–802. 24615589 10.1177/0969733014521094

[4] Abel PE. Ethics committees in home health agencies. Public Health Nurs. 1990;7(4):256–9. 2270225 10.1111/j.1525-1446.1990.tb00645.x

[5] Hestevik CH, Molin M, Debesay J, Bergland A, Bye A. Healthcare professionals’ experiences of providing individualized nutritional care for older people in hospital and home care: a qualitative study. BMC Geriatr. 2019;19(1):317. 31747884 10.1186/s12877-019-1339-0 PMC6865038

[6] Tønnessen, Nortvedt. Førde. Rationing home-based nursing care: professional ethical implications. Nurs Ethics. 2011;18(3):386–96. 21558114 10.1177/0969733011398099

[7] Fjørtoft AK, Oksholm T, Førland O, Delmar C, Alvsvåg H. Balancing contradictory requirements in homecare nursing—A discourse analysis. Nurs open. 2020;7(4):1011–9. 32587719 10.1002/nop2.473 PMC7308681

[8] Christiansen B, Bjørk IT. Godt – eller godt nok? Hvordan opplever sykepleiere idealer og realiteter i utøvelsen av yrket? Nordisk Tidsskrift Helseforskning. 2016;12(1):64. 10.7557/14.3774

[9] Gilligan C. In a different voice: psychological theory and women’s development. Massachusetts,London, England: Harvard University Press;: Cambridge; 2003.

[10] Held V. The Ethics of Care: Personal, Political, and Global. 1 ed. New York: New York: Oxford University Press; 2005. pp. viii–viii.

[11] Pettersen T. Omsorg Som Etisk teori [care as an ethics theory]. Norsk Filosofisk Tidsskrift. 2006;41(2):151–61. 10.18261/ISSN1504-2901-2006-02-05

[12] Martinsen E. Harm in the absence of care: towards a medical ethics that cares. Nurs Ethics. 2011;18(2):174–83. 21372230 10.1177/0969733010392304

[13] Brinchmann BS. Etikk i sykepleien [Ethics in nursing]. 2. Utg. ed. Oslo: Gyldendal akademisk; 2008.

[14] Heggestad AKT. Etikk i klinisk sjukepleie [Ethic in clinical nursing]. Oslo: Samlaget; 2018.

[15] Herdis A, Martinsen KM. Care and Discerning Judgement. Tidsskrift for omsorgsforskning. 2018;4:215 – 22.

[16] Gardner EH, Csikszentmihalhi M, Damon W. Good work: when Excellence and Ethics Meet. Basic Books; 2008.

[17] Heggestad AKT, Konow ASL, Christiansen B, Nortvedt P. A vulnerable journey towards professional empathy and moral courage. 2022. 10.1177/09697330221074013 PMC9289973 35225056

[18] Kleemola E, Leino-Kilpi H, Numminen O. Care situations demanding moral courage: content analysis of nurses’ experiences. Nurs Ethics. 2020;27(3):714–25. 31984838 10.1177/0969733019897780

[19] Peng M, Saito S, Guan H, Ma X. Moral distress, moral courage, and career identity among nurses: a cross-sectional study. Nurs Ethics. 2023;30(3):358–69. 36545793 10.1177/09697330221140512

[20] Kattouw CE, Wiig S. Organiseringen Av Hjemmesykepleien Kan gå Ut over sikkerhet og kvalitet [The organization of home-bsed care can go beyond safety and quality]. Sykepleien Forskning (Oslo). 2018(74391):e–74391.

[21] Helsenorge. Hjemmesykepleie og andre helsetjenester i hjemmet [Home-based care and other services in private homes] helsenorge.no: Helsedirektoratet; 2019 [cited 2021 07.09.2021]. https://www.helsenorge.no/hjelpetilbud-i-kommunene/helsetjenester-i-hjemmet/#hvem-kan-f%C3%A5-helsetjenester-i-hjemmet

[22] Birkeland A, Flovik AM. Sykepleie i hjemmet [Nursing at home]. 3. utg. ed. Oslo: Cappelen Damm akademisk; 2018.

[23] Jacobs G. Patient autonomy in home care: nurses’ relational practices of responsibility. Nurs Ethics. 2019;26(6):1638–53. 29734887 10.1177/0969733018772070

[24] Nortvedt P. Omtanke: innføring i sykepleieetikk [care: introduction to nusring ethics]. 3. Utgave. ed. Oslo: Gyldendal; 2021.

[25] Berta OG, Høgblad TN. Helt nært: en innføring i etnografisk metode [Close: an introduction to anthropological method]. Oslo: Universitetsforlaget; 2023.

[26] Fangen K. Deltagende observasjon [Participant observation]. 2. Utg. ed. Bergen: Fagbokforl; 2010.

[27] Storaker A, Heggestad AKT, Sæteren B. Ethical challenges and lack of ethical language in nurse leadership. Nurs Ethics. 2022;29(6):1372–85. 35621154 10.1177/09697330211022415 PMC9527351

[28] Munkeby H, Moe A, Bratberg G, Devik S. Ethics between the lines’ – nurses’ experiences of ethical challenges in Long-Term Care. Glob Qual Nurs Res. 2021;8:23333936211060036. 35005098 10.1177/23333936211060036 PMC8738871

[29] Ardener E, Chapman M. The voice of prophecy and other essays. Oxford: Basil Blackwell; 1989.

[30] Kvale S, Brinkmann S. Det kvalitative forskningsintervju [the qualitative research interview]. 3. utg. ed. Oslo: Gyldendal akademisk; 2015.

[31] Clifford J, Marcus GE. Writing culture: the poetics and politics of ethnography. Berkeley, Calif: University of California Press; 1986.

[32] Braun V, Clarke V. Using thematic analysis in psychology. Qualitative Res Psychol. 2006;3(2):77–101. 10.1191/1478088706qp063oa

[33] Pink S. Doing sensory ethnography. London: SAGE Publications Ltd; 2015.

[34] Braun V, Clarke V. Thematic analysis: a practical guide. Los Angeles, California: SAGE; 2022.

[35] Nurses ICN. The ICN codes of ethics for nurses 2021 [cited 2023 28.11.2023]. https://www.icn.ch/sites/default/files/2023-06/ICN_Code-of-Ethics_EN_Web.pdf

[36] Sykepleieforbund N. Yrkesetiske retningslinjer for sykepleiere [Professional ethical codes for nurses] [Guideline]. Norsk sykepleieforbund [Norwegian Nurse Society]; 2023 [cited 2023 28.11.2023]. https://www.nsf.no/etikk-0/yrkesetiske-retningslinjer-sykepleiere

[37] Helsedirektoratet H. 2018 [07.08.2023]. https://www.helsedirektoratet.no/rundskriv/helsepersonelloven-med-kommentarer

[38] Magelssen M, Førde R, Lillemoen L, Pedersen R. Etikk i helsetjenesten [Ethic in healthcare]. 1. Utgave. ed. Oslo: Gyldendal; 2020.

[39] Bjornsdottir K. The place of standardisation in home care practice: an ethnographic study. J Clin Nurs. 2014;23(9–10):1411–20. 24251979 10.1111/jocn.12412

[40] Bjornsdottir K. I try to make a net around each patient’: home care nursing as relational practice. Scand J Caring Sci. 2018;32(1):177–85. 28543618 10.1111/scs.12443

[41] Health] HDo. Utfordringsbildet Og Mulighetsrommet I den kommunale helse- og omsorgstjenesten [Challenges and opportunities in Municipal Health and Care Services]. Oslo: Helsedirektoratet [Directory of Health]; 2021.

[42] Jia Z, Kornstad T, Beyrer S, Hjemås S. Regional Variasjon i sykefraværet blnat sykepleiere og helsefagarbeidere [Regional Variation in sickness absence among nurses and Health Care assistants]. Statistic Norway; 2021.

[43] Pettersen T, Hem MH. Mature care and reciprocity: two cases from acute psychiatry. Nurs Ethics. 2011;18(2):217–31. 21372235 10.1177/0969733010392301

[44] Meld. St. 24 (2022–2023). Felleskap Og meistring. Bu trygt heime. In: omsorgsdepartement H-o, editor.

[45] Lov om helsepersonell m.v. (helsepersonelloven) [Act relating to Health Personnel etc. (Health Personnel Act)] LOV-1999-07-02-64 (1999).

[46] Rehnsfeldt A, Slettebø Å, Lohne V, Sæteren B, Lindwall L, Heggestad AKT, et al. Dignity in relationships and existence in nursing homes’ cultures. Nurs Ethics. 2022;29(7–8):1761–72. 35801831 10.1177/09697330211041739 PMC9667089

[47] Lohne V, Høy B, Lillestø B, Sæteren B, Heggestad AKT, Aasgaard T, et al. Fostering dignity in the care of nursing home residents through slow caring. Nurs Ethics. 2017;24(7):778–88. 26850071 10.1177/0969733015627297

