# Supplementary material for: Putting patients first: when home-based care staff prioritise loyalty to patients above the system and themselves. An ethnographic study
Source: BMC Med Ethics. 2024 Sep 11;25:95. doi: 10.1186/s12910-024-01094-0 (PMC11389381; doi:10.1186/s12910-024-01094-0)
Supplement: Supplementary file 1 — Supplementary Material 1 [file 12910_2024_1094_MOESM1_ESM.docx]

Observation Guide:

Purpose of the Project:

To explore the ethical challenges experienced by the staff and leaders in home-based care and how they manage these challenges. Additionally, to understand what patients and their relatives find challenging when receiving assistance from home nursing services.

Context:

Context surrounding home-based care:

• How many individuals does the home-based care care for during a shift?

• How many staff members are on duty?

• What is the size of the municipality?

• Description of the facilities where the home-based care is based

• What forums does the home-based care have for addressing ethical issues?

Context within the patient's home:

• Description of the patient's home situation - living arrangements

- Is it an apartment or a house?

- How is the house/apartment adapted for home nursing care?

- What assistive devices are available?

- What are the hygienic conditions like?

- How many people live in the household?

Observations and activities during Home-based care visits:

• How does the home-based care staff prepare for the meeting with the patient?

• What is the reason for the patient requiring home-based care? (What decisions have been made regarding the patient?)

• What does the home-based care staff do upon arrival at the patient's home?

• Are relatives present during the home visit?

• What tasks does the home-based care staff perform in the patient's home?

- What aspects are emphasized in carrying out these tasks?

- How is the interaction between the patient and the home-based care staff?

• How do the patient/relatives/home-based care staff communicate with each other?

- What topics are discussed?

- Are there any disagreements among those present (relatives, patient, and staff)?

• How long does the visit from the home-based care last?

- How does the staff in home-based care prioritize their time?

• How do the staff facilitate the patient making their own choices? (Participation)

• Are there situations where coercion is exercised, or situations that border on coercion?

• How do the staff handle situations where decisions about coercion have been made?

• How does the patient react to the assistance?

• Where are the relatives during the visit?

Observations before and after Home Visits:

• How many individuals are present during the debriefing?

• What is the atmosphere like during the debriefing, both before and after a shift?

• How are ethical issues addressed during the debriefing? And how are these discussed?

• Are there any ethical issues that frequently arise during the debriefing?

• How do the staff plan their day, and how do they distribute the patients?

• What occurs between patient visits?

• What reporting system do they use?

• How do the staff respond when discussing ethical issues that have arisen during a shift?
