# Supplementary material for: Putting patients first: when home-based care staff prioritise loyalty to patients above the system and themselves. An ethnographic study
Source: BMC Med Ethics. 2024 Sep 11;25:95. doi: 10.1186/s12910-024-01094-0 (PMC11389381; doi:10.1186/s12910-024-01094-0)
Supplement: Supplementary file 2 — Supplementary Material 2 [file 12910_2024_1094_MOESM2_ESM.docx]

**Focus Group Interview with staff home-based care.**

Introduction round where the staff introduce themselves with:

Name, professional background, duration of employment in home nursing,

• How do you experience working in home-based care?

• What are the most common ethical challenges in home-based care?

• Describe an ethical challenge you have experienced.

• How did you handle this situation?

• What do you do when you encounter ethical challenges?

• How do you discuss these challenges?

• What do you do to handle the situation as effectively as possible?

• How do you handle situations where the patient opposes health care?

• How do you facilitate the patient making their own decisions and being participative?

• Have you made any decisions about coercion? What is/was the reason for making the decision? How do/did you experience carrying out coercion?

• How do you experience the collaboration between different professional groups?

• How do you experience collaboration with the general practitioner?

• How do you experience collaboration with other services: nursing homes/short-term departments, hospitals, etc.?

**Interview Guide for key informant’s home-based care.**

Demographic Data:

Gender:

Age:

Occupational Background:

In this interview, we aim to explore your experiences of working in home-based care, what you find meaningful in your work, and the ethical challenges you encounter. There are no "right" or "wrong" answers; we are interested in your perspectives and experiences. If there are any terms that are unclear during the interview, please feel free to ask.

- Why did you choose to work in home-based care?
- Can you describe what you particularly enjoy about your job?
- Describe a typical shift in home-based care.
- Describe the most common ethical challenges you face in home-based care.
- Tell us about a situation you experienced as ethically challenging.
- How did you handle it?
- Are there any situations in which you feel unsafe at work?
- How do you handle these situations?
- Are there any situations that are particularly challenging?
- Describe a situation where a patient refused to receive healthcare. (Either by not letting you in or not wanting help from you)
- How did you/we handle this situation?
- Have you experienced making decisions about coercion?
- What was the reason, and how did you proceed?
- How do you facilitate terminally ill patients dying at home?
- What challenges do you face in these situations?
- How do you experience collaboration and communication with the general practitioner?
- How do you experience collaboration and communication with other parts of the healthcare system? (Short-term departments, allocation offices, hospitals/specialized healthcare services)
- Can you describe what it's like to meet a patient you don't know?
- What do you do the first time you visit a patient?
- Describe a situation where communication was difficult.
- Describe how you plan your workday.
- Do you plan the time with each patient yourself, or is it done for you?
- Describe situations where you have to prioritize.
- How do you prioritize your time?
- How do you prioritize between patients?
- How do you experience having to make these prioritizations?
- Can you describe what it's like to enter a patient's home for the first time?
- What considerations do you take?
- Describe what you do to maintain the patient's privacy.
- How do you experience cooperation with relatives?
- Can you describe if there are any ethics-supportive measures, such as discussion groups, ethics reflection, or other forms of processing ethical challenges?
- How do these work?
- What do you miss in terms of ethics-supportive measures?
- New and relevant questions may arise during the fieldwork, and these will be included as they arise.

**Interview Guide for key informants home-based care.**

Demographic Data:

Gender:

Age:

Occupational Background:

In this interview, we aim to explore your experiences of working in home-based care, what you find meaningful in your work, and the ethical challenges you encounter. There are no "right" or "wrong" answers; we are interested in your perspectives and experiences. If there are any terms that are unclear during the interview, please feel free to ask.

Why did you choose to work in home-based care?

Can you describe what you particularly enjoy about your job?

Describe a typical shift in home-based care.

Describe the most common ethical challenges you face in home-based care.

Tell us about a situation you experienced as ethically challenging.

How did you handle it?

Are there any situations in which you feel unsafe at work?

How do you handle these situations?

Are there any situations that are particularly challenging?

Describe a situation where a patient refused to receive healthcare. (Either by not letting you in or not wanting help from you)

How did you/we handle this situation?

Have you experienced making decisions about coercion?

What was the reason, and how did you proceed?

How do you facilitate terminally ill patients dying at home?

What challenges do you face in these situations?

How do you experience collaboration and communication with the general practitioner?

How do you experience collaboration and communication with other parts of the healthcare system? (Short-term departments, allocation offices, hospitals/specialized healthcare services)

Can you describe what it's like to meet a patient you don't know?

What do you do the first time you visit a patient?

Describe a situation where communication was difficult.

Describe how you plan your workday.

Do you plan the time with each patient yourself, or is it done for you?

Describe situations where you have to prioritize.

How do you prioritize your time?

How do you prioritize between patients?

How do you experience having to make these prioritizations?

Can you describe what it's like to enter a patient's home for the first time?

What considerations do you take?

Describe what you do to maintain the patient's privacy.

How do you experience cooperation with relatives?

Can you describe if there are any ethics-supportive measures, such as discussion groups, ethics reflection, or other forms of processing ethical challenges?

How do these work?

What do you miss in terms of ethics-supportive measures?

New and relevant questions may arise during the fieldwork, and these will be included as they arise.
